# Systemic Therapy for Metastatic Pancreatic Cancer—Current Landscape and Future Directions

**DOI:** 10.3390/curroncol31090385

**Published:** 2024-09-04

**Authors:** Daniel Netto, Melissa Frizziero, Victoria Foy, Mairéad G. McNamara, Alison Backen, Richard A. Hubner

**Affiliations:** 1 The Christie NHS Foundation Trust, 550 Wilmslow Road, Manchester M20 4BX, UK; 2 Division of Cancer Sciences, University of Manchester, Oxford Road, Manchester M13 9PL, UK

**Keywords:** pancreas, metastatic, chemotherapy, KRAS, molecular profiling

## Abstract

Pancreatic ductal adenocarcinoma (PDAC) is a significant cause of cancer-associated mortality, with a rising global incidence. A paucity of strong predictive risk factors mean screening programmes are difficult to implement. Historically, a lack of identifiable and actionable driver mutations, coupled with a relatively immunosuppressed tumour microenvironment, has led to a reliance on cytotoxic chemotherapy. The NAPOLI-3 trial has reported data supporting consideration of NALIRIFOX as a new first-line standard of care. Kirsten Rat Sarcoma Virus (KRAS) G12D mutations are present in >90% of all PDAC’s; exciting breakthroughs in small molecule inhibitors targeting KRAS G12D may open new modalities of treatment, and therapies targeting multiple KRAS mutations are also in early clinical trials. Although immunotherapy strategies to date have been disappointing, combination with chemotherapy and/or small molecule inhibitors hold promise and warrant further exploration.

## 1. Introduction

Pancreatic ductal adenocarcinoma (PDAC) is a lethal malignancy, with an estimated 5-year survival rate of just 10% [1]. The large majority of patients (80%) present with metastatic or unresectable disease; therefore, the improvement of treatments for advanced disease is a global priority [2]. Unlike many other cancers, mortality rates are rising, with cancer-related deaths projected to rise by 79% from 2018 to 2040 [3]. The incidence is higher in developed countries, owing to aetiological driving factors associated with sedentary lifestyle, Western diet, and advancing age. However, there also appears to be a predisposition towards Caucasian and Black ethnicity [2,4,5]. Patients may present with sequelae of disease, including jaundice, biliary sepsis, coeliac plexus abdominal pain, and malnourishment. The complex medical challenges this presents can often mean that clinicians are unable to deliver systemic anti-cancer therapy due to poor patient fitness. This review will focus on the management of advanced pancreatic cancer by examining past and recent clinical trial data, which together establish the current preferred systemic therapy regimens, while also discussing areas for further therapeutic development. 

## 2. Risk Factors

Age appears to be the strongest risk factor for developing PDAC, with the highest incidence in individuals aged >80 years. Due to the aging global population, the prevalence of pancreatic cancer is expected to increase [6]. Cigarette smoking is the most significant modifiable risk factor, with a relative risk (RR) of 3.0 in smokers compared with never-smokers; ex-smokers appear to be at lower risk than current smokers, highlighting the importance of encouraging smoking cessation [7]. Metabolic factors such as obesity and diabetes mellitus are also thought to contribute to the risk of developing PDAC. A history of type 2 diabetes mellitus (T2DM) corresponds with more than twice the odds of developing PDAC (odds ratio (OR) of 2.39) according to one meta-analysis, although results are variable owing to confounding factors [8]. Similarly, an increasing body mass index (BMI) is thought to result in an increased pancreatic cancer risk, with the risk highest in patients with a BMI > 35 (OR 1.62) [9]. As per the NHS Digital Health Survey for England, rates of obesity have almost doubled in the last 20 years and therefore the expected associated increase in pancreatic cancer incidence will pose a significant public health challenge.

It is estimated that as many as 10% of PDACs may derive from a genetic predisposition. Familial pancreatic cancer confers the strongest predictive risk and should be considered if two first-degree relatives have PDAC (RR of 6.4) [10]; Breast Cancer gene 2 (*BRCA 2*) mutations are the most common inherited genetic risk factor [11], while Lynch syndrome (loss of *MLH1*, *MSH2* and *MSH6* DNA repair mechanisms constituting Mismatch repair deficiency (MMRd)) confers an 8.6-fold increased risk of developing pancreatic cancer by age 70 [12]. Beyond the need for genetic counselling, such mutations may have direct therapeutic implications. The American Society of Clinical Oncology and the National Comprehensive Cancer Network (NCCN) now recommends comprehensive germline testing of all patients with pancreatic cancer after one study highlighted that up to 5.2% of the affected population harbour predisposing genetic alterations. A summary of the most pertinent risk factors is highlighted in Table 1.

A lack of robust identifiable risk factors for most affected patients has made effective risk stratification challenging and therefore no effective screening programmes have been implemented, although the ongoing Galleri trial is aiming to assess the efficacy and cost effectiveness of implementing circulating tumour DNA (ctDNA) as part of broad-based cancer screening, including pancreatic cancer.

## 3. Pathogenesis of PDAC and Challenges for Systemic Therapies

Pancreatic cancers may arise from an exocrine or endocrine lineage, although ~95% are of exocrine ductal origin [13]. PDAC is the most frequently observed cancer and is the focus of this review. PDACs most commonly develop from pancreatic intraepithelial neoplasms (PanINs) situated in the pancreatic ducts, whereas less than 10% will arise from intraductal papillary mucinous neoplasms (IPMNs) [14].

The carcinogenesis of PDAC is thought to be multifactorial, combining pressures from mutations in key genes, epigenetic alterations, and interactions with the tumour microenvironment. *KRAS* is the most commonly mutated oncogene in PDAC, although tumour suppressor genes *CDKN2A*, *TP53*, and *SMAD4* are also frequently implicated [15]. Genetic heterogeneity between the primary carcinoma and metastasis may infer that driver mutations are fundamental in early cancer formation but less important in the metastatic process. Genes that modulate cell adhesion (*CNTN5*), motility (*DOCK2*), proteolysis (*MEP1A*), and tyrosine phosphorylation (*LMTK2*) may be more critical in pancreatic tumours metastasising [16]. To date, there are no systemic therapies that are utilised in clinical practice against these targets, although *KRAS* inhibition is the focus of significant current research, as discussed below.

The pancreatic tumour microenvironment (TME) plays a critical role in the protection and subsequent propagation of PDACs. Cancer-Associated Fibroblasts (CAFs), arising from progenitor stellate cells, create a dense matrix through excessive collagen and fibronectin synthesis, resulting in a desmoplastic response [17]. CAFs may also secrete cytokines such as interleukin (IL)-6 and IL-10 that play a role in creating an immunosuppressive TME. Furthermore, an increased presence of immunomodulating M2 macrophages may inhibit T-cell antitumour response by upregulation of Programmed Death Ligand 1 (PD-L1)/Cluster of Differentiation 80 (CD80) expression [18]. A culmination of these factors allows pancreatic tumours to proliferate, evade immune surveillance and ultimately metastasise. The dense extracellular matrix (ECM) also presents a challenge for therapeutic drug penetrance and efficacy. 

## 4. Cytotoxic Chemotherapy

### 4.1. FOLFIRINOX (Leucovorin, 5-Fluorouracil (5-FU), Irinotecan, Oxaliplatin)

All three agents in this regimen have demonstrated efficacy in pancreatic cancer and therefore the combination was studied in the PRODIGE 4 phase III trial, building on promising results from phase II. Treatment-naive patients with Eastern Co-operative Oncology Group performance status (ECOG PS) 0–1 and 75 years or younger were recruited and randomised between FOLFIRINOX (oxaliplatin 85 mg/m^2^, leucovorin 400 mg/m^2^, irinotecan 180 mg/m^2^, 5-FU at a dose of 400 mg/m^2^, administered by intravenous bolus, followed by a continuous intravenous infusion of 2400 mg/m^2^ over a 46-h period, 2 weekly) and comparator arm Gemcitabine, and overall survival (OS) was the primary end point. Patients were treated for a maximum of 6 months in both arms, provided there was evidence of tumour stability or response. There was an improvement in objective response rate (ORR) in the FOFIRINOX arm compared to gemcitabine (31.6% vs. 9.4%), and OS was also significantly longer (respectively 11.1 months vs. 6.8 months). There was a significantly higher risk of grade 3 or 4 adverse events in the FOLFIRINOX arm, particularly myelosuppression and gastro-intestinal toxicity. Despite this, Quality of Life (QoL) assessments highlighted a better preserved QoL in the FOLFIRINOX arm that was statistically significant, with a 6 months hazard ratio (HR) of 0.47 (95% confidence interval (CI), 0.30 to 0.70; *p* < 0.001) [19].

When considering that this patient cohort may already experience significant frailty, modifications in the regimen were investigated to ameliorate the high risk of toxicities. This included omission of the initial 5-FU bolus and/or a dose reduction in at least one of the drugs. A meta-analysis included 11 studies that utilised modified FOLFIRINOX (mFOLFIRINOX), with the most common amendments being omission of the 5-FU bolus and variable reductions in the dose of irinotecan. Overall survival data was almost equivalent; rates of grade 3/4 adverse events were lower, particularly lethargy and gastrointestinal symptoms, providing a strong rationale for preferentially adopting the modified regimen [20]. In the PRODIGE-4 trial, the median number of cycles received in the FOLFIRINOX arm was 10, although 7% of the cohort were treated with a “stop-and-go” strategy beyond 12 cycles if there was perceived to be further benefit.

The impressive survival advantage has placed mFOLFIRINOX as a preferred first-line chemotherapy option in patients with a good PS (0–1). The safety profile highlights that this regimen required a careful patient selection to be able to realistically support patients through treatment, but positive QoL data and amendments to dosing suggest that for the right patient cohort, this is an efficacious and tolerable palliative chemotherapy. The optimum cycle length remains an unanswered question, particularly for a small group of patients that may benefit from treatment beyond 12 cycles.

### 4.2. Nab-Paclitaxel and Gemcitabine

Pre-clinical work in mouse models had demonstrated that nab-paclitaxel (albumin-bound paclitaxel) may increase intra-tumoral concentrations of gemcitabine and therefore provided rationale for clinical studies [21]. In a subsequent phase III trial (MPACT), 861 patients with a Karnofsky Performance Score (KPS) > 70 were randomised to receive nab-paclitaxel/gemcitabine vs. gemcitabine. The trial included a population of patients aged >75 years (10%). Patients were treated until disease progression. Median OS was longer with gemcitabine/nab-paclitaxel (8.5 vs. 6.7 months, HR 0.72, CI, 0.62 to 0.83; *p* < 0.001), with impressive response rates (23% vs. 7%) [22]. Superior survival demonstrated with nab-paclitaxel/gemcitabine has positioned it amongst first line standard of care options for patients with a KPS > 70. Aside from peripheral neuropathy, the side effect profile of nab-paclitaxel/gemcitabine was favourable, and the trial also supported its use in a carefully selected elderly population.

### 4.3. NAPOLI Trial Series and GENERATE Trial

As many patients inevitably face progression of disease after first-line chemotherapy, development of safe and effective second-line treatment options is a significant clinical need in PDAC. Irinotecan had already shown efficacy as incorporated in the FOLFIRINOX trials, and second-line phase II studies of irinotecan reporting a median OS of 5.2 months [23] also provided the basis for the NAPOLI-1 trial—a phase III study of second-line therapy in metastatic PDAC. Liposomal formulation has been shown to protect prodrugs, such as irinotecan, from unwanted metabolism in circulation, thus delivering a prolonged higher concentration of the active metabolite SN-38 to the tumour tissue. Preclinical studies had reported a 5.2-fold increased concentration of SN-38 at tumour sites compared with standard Irinotecan [24]. NAPOLI-1 investigated whether this translated into enhanced anti-tumour activity and prolonged survival. The trial recruited patients who had received prior treatment with gemcitabine, 5-FU and/or irinotecan, and compared liposomal irinotecan (80 mg/m^2^) + folinic acid (400 mg/m^2^) + 5-FU (2400 mg/m^2^ over 46 h) every 2 weeks vs. single agent liposomal irinotecan (120 mg/m^2^) vs. control arm of 5-FU (2000 mg/m^2^ 5-FU over 24 h) + folinic acid (200 mg/m^2^). A total of 417 patients were randomised; patients required a KPS > 70, and 88% of the intention to treat (ITT) population had received at least one prior line of systemic chemotherapy for metastatic disease. Median OS was increased with liposomal irinotecan/5-FU/folinic acid vs. control arm (6.2 vs. 4.2 months respectively; HR, 0.75; 95% CI, 0.57–0.99; *p* = 0.039)); however, there was no statistically significant benefit when comparing single agent liposomal irinotecan with the control arm (HR 0.99) [25]. These findings provided evidence to support use of this regimen as second-line palliative systemic chemotherapy.

The NAPOLI-3 trial has recently concluded and investigated the role of liposomal irinotecan in a first-line triple-drug combination treatment for metastatic PDAC. Modified FOLFIRINOX and gemcitabine/nab-paclitaxel had positioned themselves in equipoise as first-line options. The trial compared NALIRIFOX (liposomal irinotecan 50 mg/m^2^, oxaliplatin 60 mg/m^2^, leucovorin 400 mg/m^2^, and 5-fluorouracil 2400 mg/m^2^ over 46 h pump) given on days 1 and 15 of a 28-day cycle against gemcitabine/nab-paclitaxel (nab-paclitaxel 125 mg/m^2^ and gemcitabine 1000 mg/m^2^) given on days 1, 8, and 15 of a 28-day cycle, with treatment administered until disease progression or unacceptable toxicity. Median OS was superior with NALIRIFOX (11.1 vs. 9.2 months, HR 0.83, *p* = 0.036) with an impressive ORR of 41.8% (compared to 36.2% with nab-paclitaxel/gemcitabine). The published survival data mirrored results of other phase III trials that included nab-paclitaxel/gemcitabine, supporting the validity of the data. In a pre-specified subgroup analysis, the factors that seemed to predict a more favourable response with NALIRIFOX including ECOG PS 0, presence of liver metastasis and in patients over 65 years. Adverse event rates were comparable in both groups, with fewer grade 3–4 toxicities in the NALIRIFOX arm [26].

The GENERATE trial aimed to compare the most commonly used first-line chemotherapy regimens in Asia of modified FOLFIRINOX vs. S-IROX (oxaliplatin 85 mg/m^2^, irinotecan 150 mg/m^2^, S-1 80 mg/m^2^/day days 1–7, 2 weekly) vs. nab-paclitaxel/gemcitabine in a head-to-head prospective analysis. In this Japanese study, 527 patients aged 20–75 years old were recruited and randomised 1:1:1. The primary endpoint was OS in phase III. The study reported a trend towards better OS favouring nab-paclitaxel/gemcitabine (median OS respectively 14.0 vs. 13.6 vs. 17.0 months). There were expectedly fewer grade 3 toxicities with the doublet regimen compared with triplet therapy. The trial was terminated early due to futility—failing to show superiority of S-IROX; the authors therefore concluded that nab-paclitaxel/gemcitabine should be considered as the superior first-line treatment in view of the overall survival data [27]. It is important to note that a significantly higher response rate to nab-paclitaxel/gemcitabine was observed in early phase Japanese trials compared with the landmark European MPACT trial (58.8% vs. 23%). Considering that the response rates in the modified FOLFIRINOX arm of the GENERATE study were in line with other published data, the high ORR observed with nab-paclitaxel/gemcitabine is difficult to explain, and therefore results of the trial should be interpreted with caution.

Limited retrospective real world data is available to help clarify the optimal first-line chemotherapy strategy. A systematic analysis which included 14 case series and 2 cohort studies reported similar response rates for nab-paclitaxel/gemcitabine (25%) and mFOLFIRINOX (24%) with equivalent median OS (HR 0.99) [28]. Another single centre retrospective study at The Ohio State University Comprehensive Cancer Centre included 179 patients in their analysis; median OS favoured modified FOLFIRINOX (gemcitabine/nab-paclitaxel 7.5 months vs. mFOLFIRINOX 9.4 months), although selection bias cannot be excluded. Further retrospective analyses have yielded varying results, with possible survival trends favouring modified FOLFIRINOX [29,30]. In a pooled analysis of phase III trials aiming to compare efficacy and toxicity of NALIRIFOX against FOLFIRINOX and nab-paclitaxel/gemcitabine, 1372 patients were included, a large proportion of whom received nab-paclitaxel/gemcitabine (*n* = 818); progression free survival (PFS) significantly favoured NALIRIFOX (7.4 m vs. 6.4 m vs. 5.6 m), but OS was equivalent between NALIRIFOX and FOLFIRINOX (11 vs. 11.1 months) while nab-paclitaxel/gemcitabine achieved inferior OS (9.2 months). Notably, gastrointestinal toxicities were highest with NALIRIFOX [31]. It should be noted that the NAPOLI-3 trial did not include FOLFIRINOX as a comparator arm. Differences in major trials investigating the efficacy of first-line chemotherapy are shown in Table 2.

It is challenging to extrapolate definitive conclusions from retrospective non-randomised data and in practice first-line treatment selection may be based on patient factors such as frailty, differences in toxicity profile, and institution preference. Clinicians will need to consider whether to adopt NALIRIFOX as the new first-line chemotherapy regimen for metastatic pancreatic cancer or persist with the widely used modified FOLFIRINOX in patients with performance status 0–1. Furthermore, due consideration to the duration of chemotherapy courses must be given, considering that in both MPACT and NAPOLI-3, treatment continued until the point of progression or unacceptable toxicity.

### 4.4. Cisplatin + Gemcitabine

The scientific hypothesis for combining cisplatin and gemcitabine was informed from convincing pre-clinical models showing synergistic DNA damage [32,33]. A phase III Italian study compared cisplatin/gemcitabine vs. gemcitabine. Treatment was continued until progressive disease. The vast majority (84%) had metastatic disease; all patients required a KPS > 80%. There was no survival difference observed between the two arms (median OS 7.2 vs. 8.3 months). The results have been validated by other similar trials, albeit utilising different dosing schedules [34,35].

Recent data from a phase 2 study explored cisplatin + gemcitabine with or without the Poly ADP-Ribose Polymerase (PARP) inhibitor veliparib in patients with a germline BRCA or Partner and Localiser of BRCA 2 (PALB2) mutation. In the control arm, response rates were impressive, with 74.1% response in the cisplatin/gemcitabine arm and median PFS 10.1 months, greatly exceeding results observed with other first-line chemotherapies in unselected patients [36]. Interestingly, the addition of a PARP inhibitor concurrent with the chemotherapy did not appear to lead to benefit and in fact trended towards detriment. The study supports the use of cisplatin and gemcitabine as first-line treatment in preselected patients harbouring germline BRCA/PALB2 mutations with pancreatic cancer [37].

### 4.5. Gemcitabine Based Chemotherapy 

Chemotherapy for metastatic pancreatic cancer was initially trialled after clinical observation supported the idea that the administration of gemcitabine could effectively palliate cancer-related symptoms. In 1997, a phase III trial was set up to investigate the efficacy of gemcitabine vs. 5-FU. Results favoured gemcitabine, with 1-year overall survival (OS) 23.8% vs. 2% and, importantly, an improvement in palliating symptoms of pain [38]. Gemcitabine monotherapy then became the standard of care. Gemcitabine is a well-tolerated drug, with the main toxicity being myelosuppression, which is usually short-lived [39]. For this reason, gemcitabine monotherapy continues to be used in patients who are less fit (Eastern Cooperative Oncology Group [ECOG] performance status 2) or as a second-line treatment. 

In 2007, Herrmann et al. published data from a phase III randomised control trial comparing gemcitabine/capecitabine against gemcitabine. Median OS in the cohort was not statistically significant (8.4 vs. 7.2 months). However, a post hoc analysis in patients with a favourable KPS (>90) reported a significant difference in median OS favouring gemcitabine/capecitabine (10.1 m vs. 7.4 m) [40]. A further meta-analysis of three phase III trials revealed a statistically significant benefit of gem/capecitabine in prolonging OS (HR 0.86, CI 95%, 0.75–0.98; *p* = 0.02) [41]. In practice, gemcitabine/capecitabine has been largely superseded by other regimens but may be utilised in less fit patients or as a second-line therapy.

Gemcitabine/oxaliplatin (GemOx) was investigated in a phase III trial following encouraging phase 2 data and the rationale that platinums were shown to be effective against gastrointestinal tumours. There was a significant PFS benefit of GemOx compared with gemcitabine (5.8 vs. 3.7 months). However, this did not translate to a significant OS benefit (median OS in the metastatic cohort 8.5 vs. 6.7 months) which may have been confounded by frequent use of second-line platinum-based therapies in the gemcitabine arm [42]. Consequently, GemOx is infrequently used due to a lack of robust data demonstrating OS benefit.

### 4.6. FOLFOX (5-FU, Leucovorin, Oxaliplatin)

The CONKO study group investigated the efficacy of OFF (closely related to FOLFOX but differing in treatment schedule, given as folinic acid 200 mg/m^2^, fluorouracil 2000 mg/m^2^ over 24 h on days 1, 8, 15, and 22 and oxaliplatin 85 mg/m2 IV administered on days 8 and 22) as second-line chemotherapy vs. best supportive care. Patients required KPS > 60% to enrol and patients who received previous radiotherapy were excluded. A total of 168 patients were randomly assigned; median OS was significantly improved in the OFF cohort vs. BSC (4.82 vs. 2.3 months), with best response achieved being stable disease. The benefit was seen across all subgroup analysis. OFF was reasonably safe to deliver, with no grade 4 toxicities and no patients needing to stop chemotherapy due to toxicities [43]. Hence, this combination was confirmed as a second-line chemotherapy option, and FOLFOX is most commonly used due to it being a more simplified regimen.

## 5. Targeted Treatments

### 5.1. RAS Biology and RAS Inhibitors

RAS family proteins include KRAS, HRAS, and NRAS and are involved in signal transduction from upstream growth factors such as Epidermal Growth Factor Receptor (EGFR). The *KRAS* isoform specifically has been highlighted as a key oncogenic driver in pancreatic cancer. RAS protein may exist in an ON or OFF conformational state, regulated by guanine nucleotide exchange factors (GEFs) and GTP-ase activating proteins (GAPs). Mutations in codons 12 and 13 impair GAP binding or GTPase activity, leading to persistent RAS signalling, and are the most common mutations found in PDACs [44]. Codon 12 mutations are estimated to be present in up to 90% of PDACs, are associated with chemoresistance and worse OS, and as such have been at the centre of recent research [45,46,47].

Understanding the complexities of KRAS signalling has been challenging. Mouse models of PDACs with *KRAS* knockout were still able to eventually circumvent this via alternate signalling pathways, leading to cancer proliferation [48]. The recent licencing of Sotorosib and Adagrasib, KRAS G12C inhibitors, has gained much attention, particularly for their role in non-small cell lung cancer; however, G12C mutations only constitute ~3% of *KRAS* mutations in pancreatic cancer [49]. The CodeBreaK 100 phase II pan tumour trial investigating Sotorosib included 38 patients with PDAC harbouring KRAS G12C mutations and best response was measured; 8 patients had partial response and 24 patients had stable disease, while the median duration of response (DOR) was 5.7 months [50]. Other clinical trials such as KRYSTAL-1 are ongoing with the aim of assessing the efficacy of G12C inhibitors in tumour groups where G12C mutations are less frequent (non-small cell lung cancer and colorectal cancer were excluded in KRYSTAL-1) [51]. Mouse model studies of tumour cells treated with G12C inhibitors showed that a pro-inflammatory environment was created, alluding to the concept that combination with immune checkpoint inhibitors may have synergistic benefit and warrant further research [52]. Although this data is promising, development of therapies targeting the more widely observed *KRAS* mutations in PDACs will have a greater clinical impact. 

*KRAS G12D* is the most common mutation found in PDACs; therefore, efforts in pancreatic cancer have focused on G12D inhibition [53]. A novel non-covalent selective small molecule inhibitor, MRTX1133, has been shown in mouse models to induce impressive tumour regression and favourable alterations to the tumour microenvironment, correlating with significant reduction in MAP Kinase signalling pathways and an increase in tumour-infiltrating lymphocytes [54]. Translational work to bring this to early phase human trials is ongoing.

Recently, a novel multi-RAS^ON^ inhibitor RMC-6236 has been developed. Pre-clinical work had demonstrated deep and durable responses in PDACs harbouring the *KRAS G12X* mutation (X = A, D, R, S, or V—i.e., multiple different mutations involving codon 12). The phase 1 trial (NCT05379985) recruited 65 patients with PDAC and a G12X mutation, of which 49% were *KRAS G12D* mutant and 29% *KRAS G12V* mutant. Patients had received a median of three prior lines of therapy. Disease control rate in a heavily pre-treated population was an impressive 40%, with 9% of patients achieving partial response. The trial observed that a drop in variant allele frequency of *KRAS* in ctDNA was a good surrogate marker of disease control [55]. Further dose expansion cohorts are in development, but these results offer an encouraging glimpse into the future. Table 3 below provides a focused selection of current and future promising KRAS-directed therapies for metastatic pancreatic cancer, although many other early-phase trials are in progress.

RAS inhibitors have the potential to change the landscape of treatments for pancreatic cancer, and thus KRAS targeting continues to be researched rigorously.

### 5.2. Epidermal Growth Factor Receptor (EGFR) Signalling Pathways

EGFR is overexpressed in pancreatic cancer, therefore providing rationale for targeting EGFR. The first-generation small molecular EGFR inhibitor erlotinib had demonstrated efficacy in animal models and therefore progressed to being analysed in a phase III trial [58].

A total of 486 patients were randomised to receive gemcitabine plus either erlotinib or placebo in the final analysis. There was a small benefit of the addition of erlotinib to gemcitabine (median OS 6.24 vs. 5.91 months, HR 0.82, 95% CI, 0.69 to 0.99; *p* = 0.038). There were similar rates of complete and partial response (8.6% vs. 8.0% respectively). *EGFR* mutation status was not predictive of a favourable response to erlotinib [59]. The very modest clinical benefit was disappointing for the scientific community, despite over half of patients harbouring an *EGFR* oncogenic mutation, and therefore gemcitabine/erlotinib combination is not routinely utilised in clinical practice for patients with advanced pancreatic cancer.

Several early-phase studies in patients with advanced pancreatic cancer have evaluated whether Cetuximab, an anti-EGFR monoclonal antibody, may exploit EGFR signalling pathways better than erlotinib. Cetuximab has been studied in combination with chemotherapy; however, no studies have shown an improvement in OS [60,61,62]. The disappointing clinical trials of EGFR directed therapy has seen the focus diverted towards RAS inhibition.

### 5.3. PARP Inhibitors

PARP (poly (ADP-ribose) polymerase) inhibitors have shown effectiveness in patients with cancers harbouring defects in homologous recombination DNA repair pathways. The incidence of *BRCA* mutations in pancreatic cancer range from 4 to 8%, and in particular, *BRCA2* mutations confer a significant risk of developing pancreatic cancer [63]. The POLO trial, a phase III randomised, double-blinded placebo trial, investigated the role of olaparib as a maintenance treatment for patients following platinum-based chemotherapy in patients harbouring a germline *BRCA* (*gBRCA*) mutation. Patients in this study were mandated to have completed at least 16 weeks of platinum-based chemotherapy. Patients were randomly assigned to either receive olaparib 300 mg OD or placebo 4–8 weeks following the last dose of chemotherapy. This sequencing aimed to avoid the previously observed deleterious effect of giving PARP inhibitors concurrently with chemotherapy. The primary endpoint for the trial was PFS with key secondary endpoints being OS, time to second disease progression, and safety. Median duration of chemotherapy was similar in both arms. Median PFS was longer in the olaparib arm (6.7 vs. 3.7 months); however, there was no advantage in OS (19.0 and 19.2 months). Sub-group OS analysis showed that patients who received triplet chemotherapy survived longer than those receiving doublet regimens, possibly highlighting the synergistic role of chemotherapy with subsequent maintenance PARP inhibitors. It is worth noting that 2-year survival rates for olaparib vs. placebo were 37.0% vs. 27.4% [64]. The separation at the tail of the Kaplan–Meier estimator curves needs further exploration to identify a distinct subgroup of the *gBRCA* population who appear to have a prolonged response to PARP inhibitors. To date, there are no trials exploring the efficacy of PARP inhibitors in patients with somatic *BRCA* mutations in tumour samples. 

### 5.4. Other Small Molecule Inhibitors

#### 5.4.1. Neurotrophic Tropomyosin Receptor Kinase (NTRK)

*NRTK* gene fusions are rare oncogenic drivers estimated to be present in <1% of all cancers [65]. Although somatic mutations are rare, treatment with the TRK inhibitor larotrectinib may produce durable responses. In a pooled analysis of 159 adult and child patients with cancer who were treated with larotrectinib, there were two pancreatic cancer cases—one patient achieved a partial response with a median DOR of 3.5 months; however, the median DOR in the overall population was 25.9 months. The low number of pancreatic cancer cases in the study makes interpretation of the data difficult, yet it is conceivable that patients with an *NTRK1/2/3* gene fusion may derive significant benefit from NTRK inhibitors [66]. This treatment is licensed for patients where a fusion in any *NTRK* gene has been identified. 

Other actionable driver mutations in PDAC are rare but include *ALK* re-arrangement and *FGFR2* and *RET* fusions.

#### 5.4.2. CDK 4/6 Inhibitors

An early-phase trial recruited patients with advanced pancreatic and biliary tract cancers exhibiting *CDKN2A* mutations and administered Palbociclib. A total of 12 patients with pancreatic cancer were treated, largely with loss of function mutations (n = 8); median PFS was 7.2 weeks, and the trial concluded early due to futility of treatment [67].

#### 5.4.3. NOTCH 

NOTCH signalling pathways appear to be upregulated from early pathogenesis of PanINs but may also be relevant in metastatic development [68]. Experimenting with anti-NOTCH monoclonal antibody tarextumab in patients with previously untreated metastatic pancreatic cancer has failed to demonstrate a clinical benefit (median OS tarextumab vs. placebo 6.4 and 7.9 months) [69].

### 5.5. Targeting Desmoplastic Stroma

The desmoplastic stroma is critical in PDAC tumorigenesis. An excess of CAF’s, a dense collagen matrix, an immune desert, and aberrant angiogenesis contribute to therapeutic drug resistance and tumour growth. Despite this, targeting the complex TME has been challenging and wrought with scientific contradictions. Initial studies in xenograft models that focused on targeting CAF’s such as inhibition of tumour-derived sonic hedgehog (Shh) led to depletion in stromal fibroblasts but increased metastasis and poorer survival [70]. Further research has now identified protumour cancer-associated fibroblasts (iCAFs) and antitumour myofibroblastic cancer-associated fibroblasts (myCAFs) as two distinct populations [71]. Finding novel cell surface proteins unique to iCAF’s may provide further specificity for refined therapeutic inhibition. Hyaluronic acid deposition is thought to increase interstitial fluid pressure, thus contributing to drug resistance [72]. Targeting agents such as pegvorhyaluronidase alfa (PEGPH20) in combination with chemotherapy (gemcitabine/nab-paclitaxel) has been trialled in a phase II first-line study. In 116 patients, 40% achieved response, and the PFS was 6.0 months [73]. This led to a phase III trial of 494 patients randomised between nab-paclitaxel/gemcitabine/PEGPH20 vs. placebo matched arm. Although a better response rate was observed in the experimental arm (34% vs. 27%), there was no difference in OS (11.2 vs. 11.5 months) [74]. Focal adhesion Kinase (FAK) is thought to contribute to a fibrotic ECM amongst other steps in tumour growth. The phase 1b/2a ACCENT trial (NCT05355298) is exploring the use of narmaforinib, a FAK inhibitor, alongside nab-paclitaxel and gemcitabine, using ORR as the primary endpoint—results are awaited. As many cancers rely on aberrant angiogenesis for growth, the anti-VEGF inhibitor bevacizumab was tested in a phase III trial in conjunction with gemcitabine against a matched placebo arm. The majority of patients had metastatic disease. No difference in OS was achieved (5.8 vs. 5.9 months) despite its successes in other tumour sites [75]. Similar results were observed with other anti-VEGF targeting agents in large phase III trials [76]. This may be due to bypassing resistance mechanisms such as upregulation of other pro-angiogenic factors, pro-inflammatory cytokines, and selective acceleration of epithelial to mesenchymal transformation [77,78]. Galectin-1 is overexpressed in stromal tissue and may impact cell migration, epithelial-to-mesenchymal transformation, and ECM remodelling. Inhibiting Gal-1 may provide a new opportunity for treatment, although work remains at a pre-clinical stage [79]. To date, there are no approved treatments targeting the desmoplastic stroma, although selective iCAF inhibition and combination strategies are being explored preclinically and in clinical trials (NCT00655655, NCT03634332).

## 6. Immunotherapy

### 6.1. Immunosuppressive Tumour Microenvironment

*KRAS* mutations, which are present in the majority of PDACs, seem to play a vital role in orchestrating immune evasion by modulating cell surface expression of MHC class I proteins, upregulating expression of PD-L1 [80,81]. Mutant KRAS signalling may also co-ordinate the recruitment of immunosuppressive macrophages, activated stromal cells, myeloid-derived suppressor cells, and regulatory T-cells in addition to decreasing the availability of T-effector cells [82]. Paucity of dendritic cells due to WNT/β-catenin signalling also confers a “cold tumour” microenvironment [83]. In conjunction, these conditions make drug delivery to the pancreas difficult, as well as inhibiting the activity of immunotherapy in PDAC.

### 6.2. Immune Checkpoint Inhibitors (ICIs)

Although the advent of PD1/PDL-1 and CTLA-4 inhibitors has transformed the landscape of treatment for many tumours, to date, ICIs have been unsuccessful in PDAC, with trials reporting disappointing outcomes. Monotherapy with ipilimumab, an anti-CTLA4 monoclonal antibody, at 3 mg/kg, 3 weekly dosing failed to show any response in 27 patients in a phase II trial [84], whilst a phase I pan-tumour study of an anti-PD-L1 antibody included 14 patients with pancreatic cancer, with none exhibiting an objective response [85]. 

### 6.3. Mismatch Repair Deficient (dMMR) Tumours 

It is important to note rare occurrences of PDAC with dMMR, estimated to occur in <1% of cases [86]. These patients may have a favourable response to immunotherapy. The Keynote-158 study assessed the efficacy of pembrolizumab and included 22 patients with advanced pancreatic cancer with dMMR/MSI-high status who had received prior standard therapy. Objective response rate was 18.2%, with one patient showing a complete response and median DOR of 13.4 m [87]. 

### 6.4. Combined Strategies of Immunotherapy

There has been no observed benefit of combining anti-PDL-1 and CTLA-4 agents in PDAC thus far. A phase II trial enrolling patients with metastatic PDAC compared durvalumab monotherapy with combined durvalumab and tremilimumab. The combination arm demonstrated an ORR of 3.1% and median PFS of1.5 months; only one patient had a partial response lasting 24 weeks [88]. 

Due to these disappointing results, further research is required to understand the dynamic interaction between immune and tumour cells in the PDAC tumour microenvironment that facilitates the observed immune pervasive phenotypic state of pancreatic tumours. The COMBAT trial assessed combination CXC chemokine receptor 4 (CXCR4) with pembrolizumab in a phase IIa study of patients with metastatic PDAC, having demonstrated in mouse models that CXCR4 may increase T cell tumour infiltration. For patients receiving this combination as second-line treatment, median OS was 7.5 months, comparing favourably to standard of care FOLFOX and Gemcitabine [89].

### 6.5. Combined Chemo-Immunotherapy

It has been hypothesised that combining chemotherapy with immunotherapy may improve the immunogenicity of tumours, with the release of neoantigens stimulating antigen-presenting cells. A phase Ib trial has assessed anti CTLA-4 treatment ipilimumab combined with gemcitabine; only a modest median PFS was observed (2.78 months), although in a minority of responders (3/13), median DOR was 11 months and one patient exhibiting a durable response of 19.8 months. This highlights that a subsect of patients may derive substantial clinical benefit from the addition of immunotherapy [90]. Corroborative results have been observed with tremilimumab and gemcitabine, with two patients achieving a partial response [91]. 

Combined chemotherapy and PD-L1 blockade is being explored and may offer more encouraging results. A phase Ib/II study assessed gemcitabine, nab-paclitaxel, and pembrolizumab in chemotherapy-naïve metastatic pancreatic cancer, with a median OS of 15 months being better than expected and superior to than gemcitabine/nab-paclitaxel alone [92]. 

Although ICIs for patients with PDAC have disappointed in clinical trials thus far, clearly there exists a subgroup of patients beyond dMMR that derive benefit, and further translational research may provide further insights. Combination immunotherapy strategies appear to hold the most promise and warrant further trials for exploration. Beyond PD1 and CTLA-4 blockade, many other immune checkpoints are under scrutiny as potential targets for future immunotherapy in pancreatic cancer. Currently recruiting early phase trials include NCT05102721, NCT04543071, NCT06051851, and NCT04802876 and are examples of trying to combine ICIs with other small molecule inhibitors and chemotherapy. Other novel approaches include non-pharmacological manipulation of PDAC TME by electroporation in combination with ICIs (NCT03080974). 

## 7. Adoptive Cellular Strategies

Adoptive cellular treatment involves ex-vivo engineering of T cells to express either specific T-cell receptors or chimeric antigen receptors (CAR) that directly bind to tumours [93]. CAR T-cell treatment has revolutionised haematological malignancies. However, the impact in solid tumours has been largely limited to melanoma.

CAR T-cell treatment in pancreatic tumours is limited to limited case studies. Mouse models have focused on utilising surface antigens MSLN, CEA, MUC1, PSCA, CD24, HER2, and natural killer (NK) as the chimeric T-cell receptors [94]. Work published from China has highlighted two cases of metastatic pancreatic cancer who had received prior systemic therapy. CAR T-cells were manufactured to express Claudin 18.2, an isoform of CLDN18, which is highly expressed in pancreatic cancer. In patient 1, PFS was 6 months, which is impressive in comparison to standard second-line chemotherapies. Patient 2 received treatment in July 2021 and achieved a complete response; the patient remains in remission at last follow up in July 2023 [95].

Many questions remain about achieving a durable response and surrounding the optimum pre-conditioning treatment which, in haematological malignancies, has classically been fludarabine/cyclophosphamide [96]. The resistant tumour microenvironment seen in PDAC continues to pose challenges for all modalities of immunotherapy, including CAR T. In addition, the feasibility of the length of time required to engineer and administer CAR T-cell treatment is questionable in this group of patients who often have a limited prognosis.

## 8. Vaccine Therapy

Vaccine therapy utilises a cancer antigen to promote a T-cell response, thus augmenting immune rejection of the cancer. Vaccine therapy may constitute either dendritic cells, cancer peptides, or tumour cells [93]. Unfortunately, vaccine therapies, to date, have shown limited clinical activity in only small patient cohorts. 

GVAX contains irradiated pancreatic tumour cells combined with a chemokine [97]. A large phase IIb study included 169 patients with metastatic pancreatic cancer who had previously received ≥1 prior line of SACT and compared GVAX + Treg depletion with cyclophosphamide + CRS-207 (expressing the tumour-associated mesothelin, thus enhancing innate immunity) compared with physicians’ choice of single agent chemotherapy. There was no improvement in OS in the experimental arm [98].

Targeting of other peptides such as Survivin, VEGF, and WT-1 aims to exploit overexpression of these molecules in PDAC cells. Many early-phase trials have explored combining vaccine treatment with chemotherapy or checkpoint inhibitors, all with limited efficacy in metastatic cases of PDAC when compared to standard of care options [99,100,101,102,103]. 

## 9. Molecular Profiling Strategies—A Move Towards Precision Oncology

The USA NCCN published updated guidelines in 2019, with a recommendation that all patients with metastatic PDAC undergo whole exome sequencing, with up to 25% of tumours harbouring potential actionable mutations [104]. Since second-line chemotherapy provides only modest PFS benefit, it is important to embrace the growing role of precision oncology. Several large clinical trials and databases such as the Know Your Tumour (KYT) programme in the USA and PRIMUS-001 as part of PRECISION PANC platform in the UK have aimed to recruit patients and perform genomic testing panels with the hope of offering personalised cancer treatment.

The KYT trial identified 26% of patients whose cancers had actionable mutations and who received the appropriate matched targeted therapy according to molecular profiling and a multidisciplinary tumour board. In the metastatic cohort, 85% of patients receiving matched therapy had already received two prior lines of systemic therapy, indicating a heavily pre-treated population with an inherent poor prognosis. The median OS was significantly prolonged in patients receiving matched treatment vs. unmatched treatment (21.7 vs. 10.2 months). Patients with an identified *ALK* mutation received the highest benefit from crizotinib + IMRT + gemcitabine, whilst patients who had more than two lines of treatment and an ATM mutation appeared to benefit from gemcitabine + nab-paclitaxel [105]. The study acknowledged the real-world limitations that clinicians may encounter, including funding and time taken for the profiling process. Furthermore, some patients received combination treatment choices with no standardised approach, making it difficult to interpret the efficacy of each component of treatment. PRIMUS 001 trial subjects are randomised to receive either FOLFOX-A (nab-paclitaxel, oxaliplatin, folinic acid, and 5-FU) vs. AG (nab-paclitaxel and gemcitabine). Results are awaited with the hope of retrospectively analysing subgroups in the context of molecular profiling carried out at baseline, with the aim of demonstrating the effectiveness of a tailored approach to systemic therapy choice.

The COMPASS study by Aung et al. also aimed to incorporate a genomic-informed approach to patients with advanced pancreatic cancer. A clear difference in chemotherapy response was observed between “classic” and “basal-like” transcriptional subtypes, with a median DOR of 4.4 and 1.5 months, respectively. This data may suggest a limited role for chemotherapy in “basal-like” PDACs. Furthermore, the “basal-like” subtype was exclusively observed in the metastatic group, suggesting that this may be associated with a more aggressive biology or is enriched in more advanced disease stages. The study also highlighted that GATA-6 expression is inversely associated with the “basal-like” subtype and may be used as a surrogate biomarker to differentiate between the two transcriptional subtypes [106].

Molecular profiling is important to consider in patients with metastatic pancreatic cancer, although actionable mutations only constitute a minority of cases at present. Further trials are awaited to best establish how this may be uniformly adapted, with a possibility of genomic testing centres adopting focused genome sequencing on actionable mutations or where there are implications for the choice of systemic chemotherapy.

## 10. Conclusions

Chemotherapy continues to be the gold standard for most fit patients diagnosed with metastatic pancreatic cancer. Initial combination choices include NALIRIFOX and modified FOLFIRINOX, with nab-paclitaxel/gemcitabine or gemcitabine alone remaining other options to consider, with best supportive care recommended for patients with a poor performance status who are not fit for systemic treatment. The optimum duration of first-line treatment remains unclear; recent trial protocols have continued until disease progression where toxicity allows but this may be challenging to deliver in clinical practice due to economic and patient factors. Despite recent positive data from NAPOLI-3, the overall prognosis for patients treated with chemotherapy alone remains poor, with limited scope for additional lines of therapy due to reduction in performance status on disease progression. Modest incremental improvements in survival, particularly in second-line clinical trials, highlights the importance of incorporating robust quality of life analysis into trial design to ensure a realistic chance of patients receiving treatment. Recent breakthroughs in targeting commonly seen *KRAS G12D* mutations are exciting, with real potential to change the landscape of treatment for patients with metastatic pancreatic cancer; data from larger clinical trials are needed to confirm efficacy. Similarly, the increasing availability of genome sequencing in clinical practice, coupled with advancements in small molecule inhibitor development, also represents an opportunity of practising truly personalised cancer medicine for a subgroup of patients. 

## Figures and Tables

**Table 1:** Aetiological risk factors predisposing to the development of pancreatic cancer according to relative risk.

Risk Factor	Relative Risk	References
Smoking	3.0	Bosetti et al., 2012 [7]
*BRCA2* mutation	3.5	Benzel et al., 2018 [11]
Family history *	6.4	Klein et al., 2004 [10]
Lynch syndrome	8.6	Benzel et al., 2018 [12]

* Family history of two or more first degree relatives affected by pancreatic cancer.

**Table 2:** Comparison between viable first-line chemotherapy options for metastatic pancreatic cancer, including newly published data from the GENERATE trial (JCOG 1611) and NAPOLI-3.

Chemotherapy Regimen	Objective Response Rate (%)	Median Progression Free Survival (Months)	Median Overall Survival (Months)	References
Modified FOLFIRINOX	31.6	6.4	11.1	Conroy et al., 2011 [19]
	-	-	14.0	Ohba et al., 2023 [27]
Gemcitabine and nab-paclitaxel	23	5.5	8.5	Von Hoff et al., 2013 [22]
	36.2	5.6	9.2	Wainberg et al., 2023 [26]
	-	-	17.0	Ohba et al., 2023 [27]
NALIRIFOX	41.8	7.4	11.1	Wainberg et al., 2023 [26]
S-IROX	-	-	13.6	Ohba et al., 2023 [27]

Modified FOLFIRINOX: oxaliplatin, leucovorin and irinotecan. NALIRIFOX: liposomal irinotecan, 5 fluorouracil, leucovorin and oxaliplatin. S-IROX: S-1, irinotecan and oxaliplatin.

**Table 3:** Examples of current and potentially new KRAS small molecule inhibitors.

Inhibitor	Target Enzyme	Mechanism of Action	Phase of Trial Including Pancreas Patients	ORR	References
Sotorosib	KRAS G12C	His95 groove, stabilises KRAS^OFF^G12C	Phase I/II	21%	Strickler et al., 2022 [50]
Adagrasib	KRAS G12C	His95 groove, stabilises KRAS^OFF^G12C	Phase I/II	33%	Bekaii-Saab et al., 2023 [51]
RMC-6236	Cyclophilin A and KRAS	Multi-RAS^ON^ inhibition by abnormally forming a tricomplex of Cyclophilin A and KRAS	Phase I/Ib	36%	Arbour et al., 2019 [55]
BI-1701963	SOS1	Depletion of SOS1 resulting in decreased KRAS signalling	Phase I	-	Johnson et al., [56]
RMC-4630	SHP2	Locks SHP2 in inhibited state	Phase I	-	Ou et al., 2023 [57]
MRTX1133	KRAS G12D	Increases hydrophobicity, therefore disrupting Switch I and II	Pre-clinical	-	Kemp et al., 2018 [54]
